# Influence of Three Modification Methods on the Structure, Physicochemical, and Functional Properties of Insoluble Dietary Fiber from *Rosa roxburghii* Tratt Pomace

**DOI:** 10.3390/molecules29092111

**Published:** 2024-05-02

**Authors:** Yumeng Huang, Chao Li, Siyuan Zheng, Xiong Fu, Qiang Huang, Guang Liu, Qing Chen

**Affiliations:** 1 School of Food Science and Engineering, South China University of Technology, Guangzhou 510640, China; hym15960079762@163.com (Y.H.); zhengsiyuan0306@163.com (S.Z.); lfxfu@scut.edu.cn (X.F.); qiangh@scut.edu.cn (Q.H.); 2 Sericultural & Agri-Food Research Institute, Guangdong Academy of Agricultural Sciences/Key Laboratory of Functional Foods, Ministry of Agriculture and Rural Affairs/Guangdong Key Laboratory of Agricultural Products Processing, Guangzhou 510610, China; liuguang@gdaas.cn; 3 School of Food and Health, Guangzhou City Polytechnic, Guangzhou 510405, China

**Keywords:** *Rosa roxburghii* Tratt pomace, insoluble dietary fiber, modification, physicochemical characteristics, functional properties

## Abstract

*Rosa roxburghii* Tratt pomace is rich in insoluble dietary fiber (IDF). This study aimed to investigate the influence of three modification methods on *Rosa roxburghii* Tratt pomace insoluble dietary fiber (RIDF). The three modified RIDFs, named U-RIDF, C-RIDF, and UC-RIDF, were prepared using ultrasound, cellulase, and a combination of ultrasound and cellulase methods, respectively. The structure, physicochemical characteristics, and functional properties of the raw RIDF and modified RIDF were comparatively analyzed. The results showed that all three modification methods, especially the ultrasound–cellulase combination treatment, increased the soluble dietary fiber (SDF) content of RIDF, while also causing a transition in surface morphology from smooth and dense to wrinkled and loose structures. Compared with the raw RIDF, the modified RIDF, particularly UC-RIDF, displayed significantly improved water-holding capacity (WHC), oil-binding capacity (OHC), and swelling capacity (SC), with increases of 12.0%, 84.7%, and 91.3%, respectively. Additionally, UC-RIDF demonstrated the highest nitrite ion adsorption capacity (NIAC), cholesterol adsorption capacity (CAC), and bile salt adsorption capacity (BSAC). In summary, the combination of ultrasound and cellulase treatment proved to be an efficient approach for modifying IDF from RRTP, with the potential for developing a functional food ingredient.

## 1. Introduction

*Rosa roxburghii* Tratt (*R. roxburghii*), a member of the *Rosaceae* family, is commonly distributed in mountainous regions with altitudes ranging from 500 to 2500 meters in southwestern China, encompassing several provinces such as Guizhou, Sichuan, Shaanxi, Yunnan, Guangdong, and Guangxi. By the end of 2022, the cultivation area of *R. roxburghii* in Guizhou alone had reached 140,000 hectares, with an annual fresh fruit yield exceeding 300,000 tons. The fruit of *R. roxburghii* contains abundant bioactive compounds, such as polyphenols, dietary fiber, and vitamin C, which have demonstrated the ability to exert various biological activities, such as hypoglycemic, hypolipidemic, and antitumor activities [1]. However, due to its sour and astringent taste, the *R. roxburghii* fruit is often processed into juice, wine, and other products, resulting in many byproducts such as *R. roxburghii* fruit pomace (RRTP) [2,3]. The improper disposal of this fruit pomace is not only a concern for sustainability, but also contributes to environmental pollution. Nevertheless, considering that pomace is a rich source of nutrients and contains high levels of dietary fiber; it can be utilized as a natural dietary fiber source [4].

Dietary fiber (DF) refers to carbohydrate compounds present in plants that cannot be digested or absorbed within the human small intestine but can be utilized by the gut microbiota, thereby contributing to enhanced gut health [5]. DF can be divided into two primary categories: soluble dietary fiber (SDF), and insoluble dietary fiber (IDF). Compared to IDF, SDF from plants exhibits better blood glucose regulation and can reduce lipid digestion [6]. To amplify the functional attributes of DF, a prevalent focus in food science research involves modifying IDF to increase the proportion of SDF [7]. The natural DF content in *R. roxburghii* fruit residue primarily consists of IDF, which limits its physiological functionality as dietary fiber and hampers its application in food. Therefore, it is essential to identify an appropriate strategy to modify IDF to promote its conversion to SDF and improve its physical and chemical properties. 

The use of ultrasonication has become widespread in treating plant biomass fibers and has steadily gained popularity as a convenient and environmentally friendly approach over time. For example, Huang et al. [8] delved into the impacts of ultrasound treatment on garlic straw, achieving favorable outcomes. Meanwhile, Zhang et al. [9] combined ultrasonication with microwave and high-pressure homogenization techniques to treat sugarcane fibers. Moreover, ultrasound has been utilized to alter the structural features and thermal properties of dietary fibers from citrus, apple, oat, and pea sources [10]. Ultrasonic processing effectively destroys plant cell walls and loosens the dense spatial network structure, resulting in better dispersion of dietary fiber in solvents and converting certain water-insoluble components into water-soluble components [11]. Simultaneously, enzymes, especially cellulase, offer a sustainable and biodegradable means to transform cellulose molecules into simpler sugars like β-glucose or shorter polysaccharides. Zhang et al. [12] found that cellulase-based enzymatic treatment can remodel the fiber network, reduce cellulose and hemicellulose content, and increase the SDF content, resulting in enhanced physiological functions such as glucose/cholesterol adsorption capacity of DF derived from potato residue. The synergistic combination of ultrasonication and cellulase enzyme holds promise for further enhancing the functional properties of dietary fibers derived from RRTP, but there is limited research on their combined effects.

Therefore, in this study, IDF, as the main component of DF from RRTP, was modified using ultrasound, cellulase, and a combination of both. The structural, physicochemical, and functional properties of the modified IDFs were evaluated, providing experimental support for the modification and utilization of IDF from RRTP in food and nutritional applications.

## 2. Results and Discussion

### 2.1. Chemical Composition

The impacts of ultrasound, cellulase, and a combination of ultrasound and cellulase methods on the basic compositions of RIDF are shown in Table 1. Compared with the raw RIDF, the contents of ash, fat, protein, and TDF in U−RIDF, C−RIDF, and UC−RIDF did not change much. However, the SDF content showed a significant increase from the original 3.92% (RIDF) to 7.86% (U−RIDF), 8.23% (C−RIDF), and reached a peak at 9.15% in UC−RIDF (*p* < 0.05). This increase coincided with a decrease in IDF content. Such a remarkable transformation indicated that cellulase and ultrasound technology could effectively destroy glycosidic bonds, release active components, and convert IDF into SDF. Particularly, UC−RIDF exhibited the highest content of SDF, registering a 2.3-fold increase compared to the raw RIDF. This could be attributed to the combined treatment conditions effectively breaking down the internal structure of the cell walls, thereby enhancing the conversion rate of insoluble components. Ultrasonic waves act first, disrupting intermolecular bonds in fibrous tissues, rendering IDF more susceptible to subsequent enzymatic action. Subsequently, cellulase enzymes cleave cellulose chains in the fiber tissue of IDF, thereby promoting the relaxation of the bonds between cellulose molecules and converting IDF into SDF [13]. This elevated SDF content holds significant potential from both the food processing and health implications points of view [14], as SDF not only contributes to the sensory and textural attributes of the final product, but also provides valuable health benefits. Although other methods, such as cross-linking and carboxymethylation, have demonstrated the ability to increase SDF content, as evidenced by boosting the SDF percentage in modified sorghum dietary fiber from an initial 2.45% to 3.85% and 7.70%, respectively [15], but their increments pale in comparison to those achieved in the present study. Therefore, the combination of ultrasonic treatment with cellulase modification technology emerges as an efficient method for modifying RIDF, promising broad application prospects.

### 2.2. Structural Analysis

#### 2.2.1. Surface Morphology

**Figure 1:**
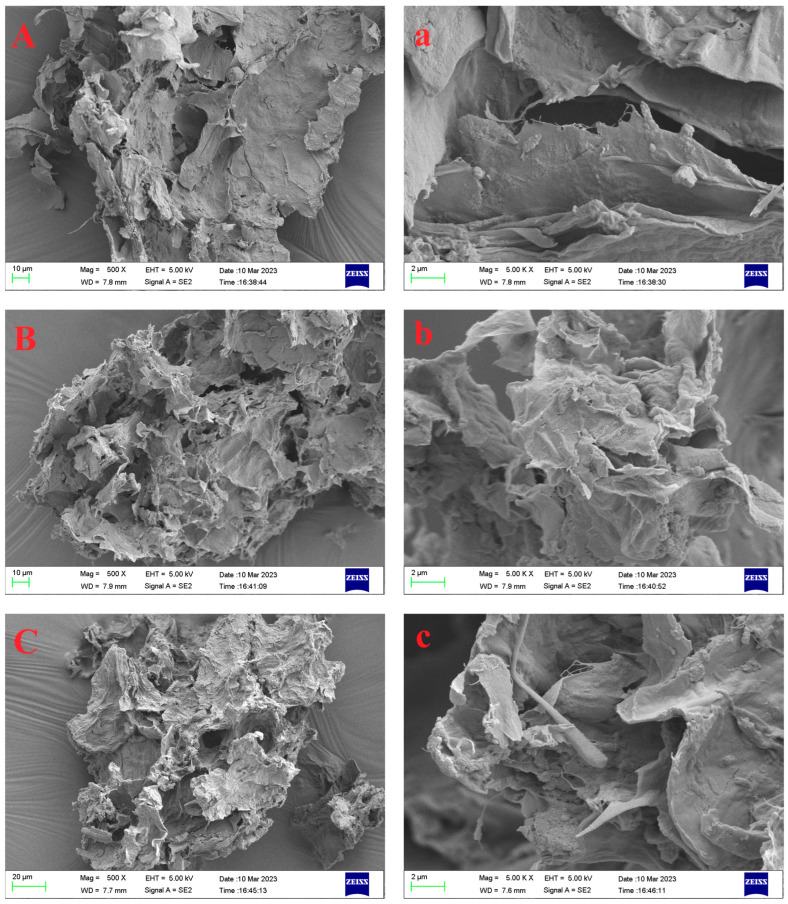

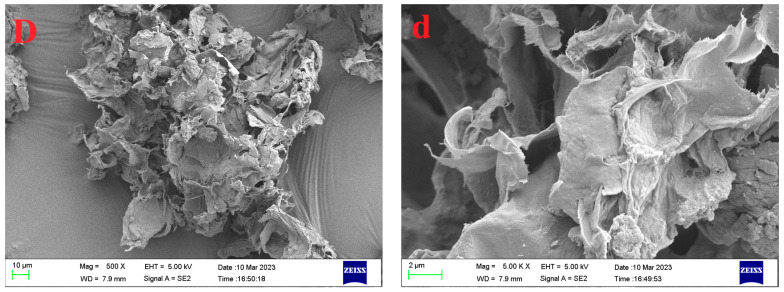
Scanning electron micrographs of RIDF (**A**,**a**), U−RIDF (**B**,**b**), C−RIDF (**C**,**c**), and UC−RIDF (**D**,**d**). Above pictures: 500 × magnification; below pictures: 5000× magnification. RIDF: *Rosa roxburghii* Tratt pomace insoluble dietary fiber; U−RIDF: RIDF modified by ultrasound; C-RIDF: RIDF modified by cellulase; UC−RIDF: RIDF modified by ultrasound-assisted cellulase.

The morphological characteristics of the experimental samples were observed using SEM at magnification times of 500× and 5000×. As depicted in Figure 1, the raw RIDF exhibited a smooth surface and a dense structure, consistent with the findings from a previous study on RIDF [16]. In contrast, the three modified RIDFs exhibited increased surface irregularities, characterized by the presence of more pronounced wrinkles and a slightly fluffy texture. This can be primarily due to the powerful mechanical forces and cavitation effects exerted by ultrasonic treatment, leading to the degradation of fibers and hemicelluloses and the formation of a loose surface structure. Additionally, the enzymatic hydrolysis of cellulose disrupts the intricate fiber network, thereby enhancing the surface roughness and porosity of the IDF samples. A looser structure typically implies that there are more voids and pores between the fibers, which is beneficial for their physicochemical properties, such as WHC [17]. 

#### 2.2.2. FTIR Spectroscopy

**Figure 2:**
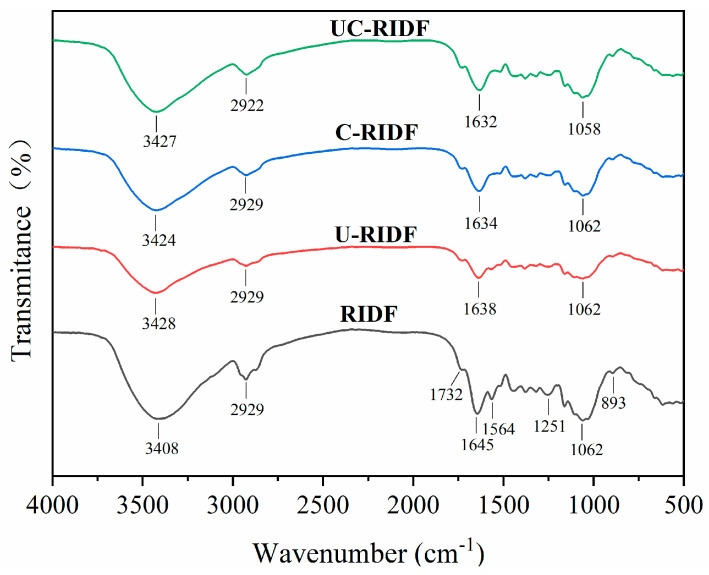
FTIR spectra of RIDF, U−RIDF, C−RIDF, and UC−RIDF. RIDF: *Rosa roxburghii* Tratt pomace insoluble dietary fiber; U−RIDF: RIDF modified by ultrasound; C−RIDF: RIDF modified by cellulase; UC−RIDF: RIDF modified by ultrasound-assisted cellulase.

FTIR spectroscopy served as a tool to characterize the chemical functional groups and identify structural changes within the substances. As depicted in Figure 2 the raw and modified RIDFs had similar spectral patterns. The absorption peak near 2929 cm^−1^ belonged to the characteristic peak of C−H absorption vibration. The absorption peak near 1732 cm^−1^ could be associated with the stretching vibration of the C=O bond, mainly from carboxylic acid. However, changes in the wave numbers and intensity of certain characteristic bands revealed the influence of modification treatments on the RIDF’s structure. The absorption peak near 1645 cm^−1^ corresponded to the stretching vibration absorption of C=O, indicating the presence of amide group [18]. Notably, the adsorption peak intensity of UC−RIDF at 1632 cm^−1^ was stronger than that of the other modified IDF groups, indicating an increased uronic acid content. Furthermore, the presence of uronic acid positively influenced the adsorption capacity of UC−RIDF. [19]. The spectral band around 3408 cm^−1^ represented the O–H stretching vibration peak. Ultrasonic, cellulase, and ultrasound-assisted cellulase treatments caused a redshift of the spectrum to near 3427 cm^−1^, accompanied by decreased peak intensity. These results demonstrated that ultrasonic treatments effectively broke down cellulose and hemicellulose, while the action of cellulase further disrupted the hydrogen bonds connecting these molecules. This facilitated the exposure of functional groups, potentially altering their physical and chemical properties [20]. The absorption peaks around 1062 cm^−1^ and 1163 cm^−1^ were associated with the C−O stretching vibrations of the sugar ring C−O−C in cellulose and lignin [21]. It was observed that the intensity of these two peaks decreased after modification, suggesting that the modification process disrupted the intermolecular forces in cellulose and altered the structure of IDF.

### 2.3. WHO, OHC, and SC Analysis

The water−holding capacity (WHC) of IDF pertains to its ability to retain water under external centrifugal force or compression. A high WHC can promote the volume of excrement and accelerate the rate of defecation, thereby abbreviating the retention time of residual foodstuffs within the gastrointestinal tract, which is effective in preventing the development of intestinal diseases [22]. As shown in Table 2**,** the WHC values of U−RIDF (10.67 g/g), C−RIDF (12.1 g/g), and UC−RIDF (11.44 g/g) were higher than that of raw RIDF (10.21 g/g), representing an increase of approximately 4.5%, 18.5%, and 12.0%, respectively. This increase is attributed to the higher SDF content and the loose structure of the modified IDF samples [23]. SDF can form gel-like substances in an aqueous solution, and the porous and laminated structure can expose more hydrophilic groups and increase the specific surface area, resulting in enhanced water retention. In contrast, a previous study that modified the RIDF via high-pressure treatment reported a WHC value of approximately 8.0 g/g [24]. This indicates that the modification techniques employed in this study are highly effective in enhancing the WHC of RIDF, potentially due to the unique structural alterations and increased SDF content in the modified samples.

The oil-binding capacity (OHC) of IDF is crucial for preventing oil loss during food processing and aiding in the reduction of serum cholesterol levels by effectively binding to dietary fat within the human digestive system [25]. The surface of the fiber contains numerous lipophilic groups, which aid in the physical entrapment of oil. As shown in Table 2, the OHC values of U−RIDF, C−RIDF, and UC−RIDF were significantly higher compared to RIDF, with increases of approximately 69.6%, 86.4%, and 84.7%, respectively. These results align with the OHC outcomes observed in the context of successive enzymatic-ultrasonic treatment of DF derived from sisal waste and *Moringa oleifera* stem [26].

The swelling capacity (SC) measures the change in the overall volume of dietary fiber and water throughout a specific time interval. Generally, the existing literature on mechanically and enzyme-treated dietary fiber compounds mostly reports an increase in SC [13,27,28]. Ultrasound treatment increases porosity and opens up the fiber matrix through cavitation effects, while enzyme treatment allows water molecules to bind to exposed hydrophilic groups and incorporate them into the fiber matrix, enhancing swelling capacity. In this study, the modification of IDF samples significantly increased their SC. In addition, the ultrasound-cellulase treatment resulted in the most substantial increase, from 5.42 mL/g to 10.37 mL/g, representing a remarkable increase of approximately 91.7% (Table 2).

### 2.4. Functional Analysis 

#### 2.4.1. Nitrite Ion Adsorption Capacity (NIAC)

**Figure 3:**
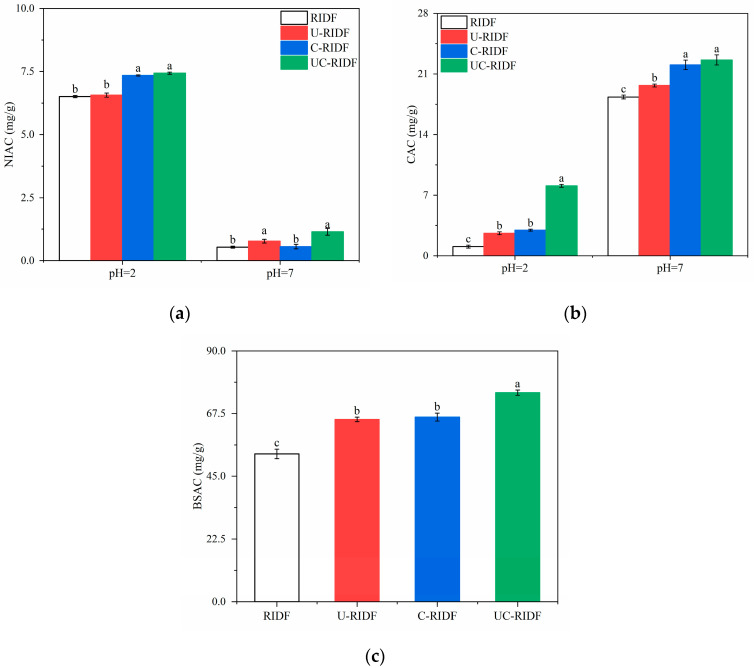
Functional properties of RIDF, U−RIDF, C−RIDF, and UC−RIDF. (**a**) Nitrite ion adsorption capacity (NIAC); (**b**) cholesterol adsorption capacity (CAC); (**c**) bile salt adsorption capacity (BSAC). Values are expressed as the mean ± SDs (*n* = 3). Different lowercase letters indicate remarkable differences between groups at *p* < 0.05. RIDF: *Rosa roxburghii* Tratt pomace insoluble dietary fiber; U-RIDF: RIDF modified by ultrasound; C−RIDF: RIDF modified by cellulase; UC−RIDF: RIDF modified by ultrasound-assisted cellulase.

Nitrite ions are commonly added to cured meat products to impart distinct flavors and colors and to inhibit the growth of pathogens. However, nitrite ions are easy to react with secondary and tertiary amines naturally present within the meat, leading to the formation of N-nitrosamine compounds, which are known to have carcinogenic effects [29]. The adsorption capacity of IDF to nitrite constitutes a crucial property that helps reduce the toxicity of cured meat products to humans. The NIAC of RIDF before and after modification treatment is shown in Figure 3a. To simulate gastric and intestinal conditions, the NIAC of RIDF samples was measured at pH 2.0 and 7.0, respectively. The results revealed that the NIAC values were higher at pH 2.0 compared to pH 7.0, indicating that IDF primarily adsorbs nitrite ions in the stomach. At a pH of 2.0, the NIAC of U-RIDF showed no significant difference compared to that of raw RIDF. However, the NIAC of C−RIDF and UC−RIDF exhibited a significant rise. Among them, UC−RIDF exhibited the highest NIAC. This enhanced adsorption capacity could be attributed to surface charge and increased exposure of polar sites on the surface of the modified RIDF particles [30]. 

#### 2.4.2. Cholesterol Adsorption Capacity (CAC)

The CAC acts as a pivotal metric for evaluating IDF’s capability to absorb lipophilic compounds in simulated gastric (pH 2.0) and intestinal (pH 7.0) environments. As depicted in Figure 3b, the CAC of IDF samples was higher under neutral conditions (pH 7.0) compared to acidic conditions (pH 2.0), implying that the adsorption of cholesterol by IDF primarily occurs in the intestine. The adsorption mechanism of the acid and alkaline systems on cholesterol can be explained from two perspectives. Firstly, the abundance of H^+^ ions in acidic conditions imparts a partial positive charge to both cholesterol molecules and IDF, resulting in electrostatic repulsion and subsequently reducing the CAC. Secondly, certain side chain groups present in IDF may dissociate at higher pH levels, allowing for the increased chelation of cholesterol molecules and an enhancement of the CAC. The active groups on the surface of IDF can adsorb cholesterol, thereby reducing cholesterol and plasma levels in the liver and preventing conditions such as gallstones and coronary atherosclerosis [31]. Interestingly, the modified RIDFs exhibited improved CAC compared to the raw RIDF. Among the modified RIDFs, UC−RIDF demonstrated the highest CAC values with 8.08 mg/g at pH 2.0 and 22.61 mg/g at pH 7.0, respectively. This enhancement can be attributed to the degradation of macromolecular IDF into smaller fragments and the increased SDF content caused by the combined modification using ultrasound and cellulase. 

#### 2.4.3. Bile Salt Adsorption Capacity (BSAC)

The bile acid contained in bile can emulsify fat in the intestine and promote fat hydrolysis and body absorption. IDF can effectively bind bile salts [32]. As shown in Figure 3c, the trend of BSCA was similar to that of CAC. The adsorption capacity of raw RIDF to sodium cholate was 53.06 mg/g, which was increased to 65.40, 66.23, and 74.96 mg/g by ultrasound, cellulase, and ultrasound–cellulase combined treatment, respectively. It could be evidently seen that UC−RIDF exhibited the highest adsorption capacity for sodium cholate. This result could be attributed to the structural changes induced by the ultrasound-cellulase combination, which created interparticle voids that effectively bound and adsorbed sodium cholate molecules.

### 2.5. Total Phenolic Content (TPC)

Polyphenols encompass a diverse range of compounds found in fruit constituents. These plant-derived phenolic compounds can be categorized into free polyphenols (FP) and bound polyphenols (BP) based on their different affinities for the cell wall matrix in plants. The combination force between FP and the plant cell wall matrix is relatively weak, allowing them to be readily released in the upper digestive tract, thereby exerting health-promoting effects. Conversely, BP forms a strong bond with the plant cell wall matrix, resisting digestion in the stomach and small intestine, and ultimately reaching the lower gastrointestinal tract of humans, where it is released from the plant cell wall matrix and further fermented into phenolic acid by intestinal microbiota, contributing to the formation of an antioxidant environment [33]. As shown in Table 3, after modification, the contents of FP, BP, and TP of RIDFs were significantly reduced, all of which decreased the FPC value most significantly. Among the three modification methods, the combined treatment of ultrasound and cellulase resulted in the most significant decrease in both FPC and BPC values. This phenomenon could be attributed to the fact that these modification treatments, particularly the ultrasound–cellulase combined treatment, facilitated the release of BP by effectively disrupting the fiber structure and enhancing fiber porosity, thereby reducing the phenolic content remaining in the extracted IDF. A previous study indicated that litchi pomace dietary fiber modified by cellulase and laccase exhibited a reduction in TPC, which could be largely attributed to the prolonged hydrolysis treatment and the use of 95% ethanol precipitation during the EH modification process [34]. 

### 2.6. Antioxidant Activities

The antioxidant activities of IDF are assessed through its capacity to scavenge radicals. As shown in Table 3, the modified RIDFs, particularly UC-RIDF, exhibited lower free radical scavenging activity than raw RIDF. This can be attributed to the significant reduction in polyphenolic compound content of RIDF after modification, particularly the combination of ultrasound and cellulase treatment. Previous studies have also reported a decline in the antioxidant activity of litchi pomace IDF after enzyme exposure, which is related to the reduced polyphenol content after enzyme treatment. The presence of various polyphenols in the fibers forms glycosidic complexes with flavonoid glycosides or phenolic acids, thereby influencing the antioxidant activity of the fibers [35]. The combined results of this study and previous research underscore the significance of BP in determining the antioxidant capacity of IDF.

## 3. Materials and Methods

### 3.1. Materials and Chemicals

RRTP was obtained from Guizhou Xinyang Agricultural Science and Technology Development Co., Ltd. (Bijie, Guizhou province, China). Thermostable α-amylase (enzyme activity: 40,000 U/g), neutral protease (enzyme activity: 100 U/mg), and cellulase (enzyme activity: 50 U/mg) were purchased from Yuanye Bio-Technology Co., Ltd. (Shanghai, China). DPPH and ABTS were purchased from Sigma-Aldrich (St Louis, MO, USA). 

### 3.2. Extraction of Insoluble Dietary Fiber from RRTP

The IDF from RRTP was extracted following a reported procedure with some modifications [36]. Briefly, RRTP was pulverized into a fine powder using a laboratory mill and then passed through a 60-mesh sieve. Subsequently, the powdered RRTP was defatted with *n*-hexane until the supernatant became colorless. The resultant residue was obtained through centrifugation at 5000× *g* for 5 min, followed by drying at 45 °C for 24 h. After drying, the defatted sample was blended with 20 times the volume of deionized water, and the pH of the solution was changed to 5.5. Thereafter, 1% thermostable α-amylase (*w*/*w*) was added, and the reaction solution was shaken at 95 °C for 60 min. Following this, the pH was adjusted to 7.0, and 0.05% natural protease (*w*/*w*) was added. The mixture was then incubated at 50 °C for 120 min. To terminate the enzymatic process, the slurry was subjected to a heat treatment at 100 °C for 5 min. After cooling, it was centrifuged at 5000× *g* for 10 min to separate the precipitate. The precipitate was washed twice with 95% ethanol (*v*/*v*), then rinsed three times with deionized water, and finally subjected to vacuum freeze-drying to obtain the RIDF.

### 3.3. Modification of Insoluble Dietary Fiber

#### 3.3.1. Ultrasonic Modification

The obtained IDF was modified following a method with slight modifications [8]. The RIDF was dispersed in deionized water (1:20, *w*/*v*) and subjected to treatment using an ultrasonic disruptor (JY92-IIN, Ningbo Scientz. Biotechnology Co., Ningbo, China) for 30 min with intermittent ultrasonication (2 s on, 3 s off) at a power of 360 W. Subsequently, fourfold volumes of 95% ethanol solution was added for alcohol precipitation. The mixture was allowed to stand for 12 h, followed by centrifugation at 5000× *g* for 10 min to collect the sediment. The ultrasonically modified insoluble dietary fiber (U−RIDF) was obtained using vacuum freeze-drying.

#### 3.3.2. Cellulase Modification

Enzymatic treatment was conducted on the IDF from RRTP following the method described by Wang et al. [37]. According to the ratio (1:20 (*m*/*v*)) of RIDF to solution, 0.3% cellulase (*w*/*w*) was added to RIDF, and the resulting solution was stirred and incubated at 50 °C for 60 min. To halt the enzymatic process, the mixture was subsequently heated at 100 °C for 5 min, followed by cooling to ambient temperature. Upon reaching room temperature, the mixture was subjected to alcohol precipitation by the addition of fourfold volumes of 95% ethanol solution. The suspension was allowed to stand for 12 h, after which it was centrifuged at 5000× *g* for 10 min to separate the precipitate. The cellulase-modified insoluble dietary fiber (C−RIDF) was obtained using vacuum freeze-drying.

#### 3.3.3. Ultrasonic-Assisted Cellulase Modification

Ultrasonic treatment was carried out as described in Section 3.3.1, followed by cellulase degradation at 50 °C for 60 min. Specifically, the RIDF was mixed with distilled water (1:20, *w*/*v*) and sonicated using an ultrasonic cell disruptor at 360 W for 30 min. Subsequently, 0.3% cellulase (*w*/*w*) was introduced, and the blend was allowed to incubate for 60 min at 50 °C. The enzymatic reaction was terminated by heating the resulting slurry at 100 °C for 5 min. Upon reaching room temperature, the suspension was subjected to alcohol precipitation by the addition of fourfold volumes of 95% ethanol solution. The mixture was allowed to stand for 12 h, followed by centrifugation at 5000× *g* for 10 min to collect the sediment. Finally, vacuum freeze-drying was used to obtain the ultrasound-assisted cellulase modification of insoluble dietary fiber (UC−RIDF).

### 3.4. Chemical Composition

The Association of Official Analytical Chemists (AOAC) method was employed to determine the basic components of the samples. Specifically, the AOAC method 991.43 was used to assess the contents of SDF, IDF, and TDF [38]. Furthermore, the AOAC methods 942.05, 925.09, 955.04, and 920.39 were utilized to measure the contents of ash, moisture, crude protein, and fat in the samples, respectively [39]. 

### 3.5. Structural Characteristics

#### 3.5.1. Scanning Electron Microscopy (SEM)

The morphological features of the samples were examined using a SEM (Merlin, Zeiss, Jena, Germany). The freeze-dried IDF samples were evenly distributed onto a sample holder and subsequently coated with gold using an ion-sputtering coater. Representative images were observed at an acceleration voltage of 20 kV and at a magnification of 500× and 5000×, respectively.

#### 3.5.2. Fourier Transform Infrared Spectroscopy (FTIR)

Each freeze-dried IDF sample was mixed with KBr powder in a 1:100 ratio into an agate mortar and ground thoroughly under infrared irradiation. Thereafter, the mixture was pressed to form a uniform transparent tablet of 1–2 mm thickness and analyzed using an FTIR spectrometer (Nicolet IS50-Nicolet Continuum, Thermo Fisher Scientific, Waltham, MA, USA). During analysis, the instrument scanned over a range extending from 4000 to 400 cm^−1^ with a resolution of 4 cm^−1^, and executed 32 consecutive scans.

### 3.6. Physicochemical Properties

#### 3.6.1. Determination of Water-Holding Capacity (WHC)

The WHC of the samples was measured following a previously reported method [40] with slight modifications. Briefly, 0.2 g of the IDF sample (m) was transferred into a 50 mL centrifuge tube (m_0_) and mixed with 20 mL of deionized water for 24 h at room temperature. After the treatment, the mixture was centrifugated at 5000× *g* for 10 min. The supernatant was then removed, and the tube and residue (m_1_) were weighed. The WHC was calculated using the following Formula (1):

(1) $$W\mathrm{HC}\left(g/g\right)=\frac{\left(m_{1}-m_{0}-m\right)}{m}$$

where m_0_ is the weight of the centrifuge tube, m is the weight of the dry sample, and m_1_ is the weight of the wet sample.

#### 3.6.2. Determination of Oil–Holding Capacity (OHC)

The OHC of the samples was measured according to a previously reported method [40]. Briefly, 0.2 g of the IDF sample (m) was transferred into a 50 mL centrifuge tube (m_0_) and mixed with 15 mL peanut oil for 24 h at room temperature. After the treatment, the oil was removed by centrifuging the mixture at 5000× *g* for 20 min. The weight of the tube and residue was recorded as m_1_. The OHC was calculated using the following Formula (2):

(2) $$\mathrm{OHC}\left(g/g\right)=\frac{\left(m_{1}-m_{0}-m\right)}{m}$$

where m_0_ is the weight of the centrifuge tube, m is the weight of the dry sample, and m_1_ is the weight of the wet sample.

#### 3.6.3. Determination of Swelling Capacity (SC)

The SC of the samples was measured following a previously reported method [41] with slight modifications. Briefly, 0.1 g of the IDF sample (m) was put in a 10 mL measuring cylinder and mixed with 5 mL of deionized water. The initial volume of the IDF sample was recorded as V_0_. After stirring, de–foaming, and allowing the mixture to stand for 24 h at room temperature, the final volume of the IDF sample was recorded as V_1_. The SC was calculated using the following Formula (3):

(3) $$\mathrm{SC}\left(\mathrm{mL}/g\right)=\frac{\left(V_{1}-V_{0}\right)}{m}$$

where V_1_ is the volume of the swollen sample (mL), V_0_ is the volume of the dry sample (mL), and m is the weight of the dry sample (g).

### 3.7. Functional Properties

#### 3.7.1. Determination of Nitrite Ion Adsorption Capacity (NIAC)

The NIAC of the samples was determined using a slightly modified version of the previously reported method [42]. In total, 0.2 g of the IDF sample (m) was thoroughly mixed with 20 mL of sodium nitrite solution (100 μmol/L). The mixture underwent pH adjustment to mimic the acidic conditions of the stomach (pH 2.0) and the neutral conditions of the small intestine (pH 7.0). Incubation was then carried out at 37 °C for 2 h. Subsequently, the supernatant was isolated through centrifugation at 5000× *g* for 20 min. The concentration of nitrite ions in the supernatant was quantified using the N-1-naphthylethylenediamine dihydrochloride method. The NIAC of different samples was calculated using the following Formula (4):

(4) $$\mathrm{NIAC}\left(\mathrm{mg}/g\right)=\frac{\left(m_{0}-m_{1}\right)}{m}$$

where m_0_ is the weight of NaNO_2_ before adsorption, m_1_ is the weight of NaNO_2_ after adsorption, and m is the weight of the dry IDF sample.

#### 3.7.2. Determination of Cholesterol Adsorption Capacity (CAC)

The CAC of the IDF sample was determined using a slightly modified version of the previously reported method [43]. Fresh egg yolk was diluted with 9 volumes of deionized water and homogenized through stirring. Subsequently, 0.1 g of the IDF sample (m) was incorporated into 15 mL of the diluted egg yolk solution and mixed evenly. The mixture underwent pH adjustment to mimic the acidic conditions of the stomach (pH 2.0) and the neutral conditions of the small intestine (pH 7.0). The mixture was gently agitated at 37 °C and 150 rpm for a duration of 2 h. Then, the supernatant was collected using centrifugation at 5000× *g* for 20 min. The cholesterol content in the supernatant was measured using the o-phthalaldehyde method. The CAC of different samples was calculated using the following Formula (5):

(5) $$\mathrm{CAC}\left(\mathrm{mg}/g\right)=\frac{\left(m_{0}-m_{1}\right)}{m}$$

where m_0_ is the initial weight of cholesterol, m_1_ is the weight of cholesterol after adsorption, and m is the weight of the dried IDF sample.

#### 3.7.3. Determination of Bile Salt Adsorption Capacity (BSAC)

The BSAC of the IDF sample was determined using a slightly modified version of the previously reported method [44]. In total, 0.25 g of IDF sample (m) was thoroughly mixed with 25 mL of sodium cholate (1 mg/mL). The pH of the mixture was adjusted to 7.0. The mixture was agitated at 150 rpm at 37 °C for 120 min. Following centrifugation at 5000× *g* for 10 min, the resultant supernatant was gathered and analyzed for sodium cholate concentration utilizing the furfural colorimetric method. The BSAC of different samples was calculated using the following Formula (6):

(6) $$\mathrm{BS}\mathrm{AC}\left(\mathrm{mg}/g\right)=\frac{\left(m_{0}-m_{1}\right)}{m}$$

where m_0_ is the initial weight of sodium cholate, m_1_ is the weight of sodium cholate after adsorption, and m is the weight of the dried IDF sample.

### 3.8. Measurement of Total Phenolic Content (TPC)

Each IDF sample (2 g) was extracted with 30 mL of 70% ethanol for 2 h, followed by centrifugation at 5000× *g* for 5 min to separate the mixture. This operation was repeated twice more, and the supernatant was collected to obtain free phenolics (FP). The precipitated fraction was subsequently subjected to hydrolysis by adding 25 mL of 4 M NaOH and incubating at room temperature for 2 h with continuous shaking in a nitrogen atmosphere. After hydrolysis, the solution was adjusted to pH 2.0 and extracted six times with ethyl acetate. The ethyl acetate phase was pooled and subjected to rotary evaporation to obtain bound phenolics (BP). 

The total phenolic content (TPC) was determined using the Folin–Ciocalteu reagent method [45]. Briefly, 100 μL of the supernatant was combined with 400 μL of distilled water and 100 μL of Folin–Ciocalteu reagent. Subsequently, the mixture was vigorously vortexed to ensure thorough mixing. After standing for 6 minutes, 1 mL of 7% sodium carbonate and 800 μL of distilled water were added to the solution. The solution was mixed again using vortexing without light. After an incubation period of 90 min, the absorbance at 760 nm was measured using a microplate reader. Gallic acid served as a reference standard, and the results were reported as milligrams of gallic acid equivalent per 100 grams of IDF (mg GAE/100 g). 

### 3.9. Determination of Antioxidant Activity

The assays of DPPH and ABTS radical scavenging capacity were performed according to the previously reported methods [46,47]. The absorbance for both the DPPH and ABTS assays was measured using a microplate reader at wavelengths of 515 nm and 734 nm, respectively. The results were expressed as micromole Trolox equivalents per gram of dry weight sample (μmol TE/g, DW). The standard curve was drawn using a range of Trolox concentrations (25, 50, 100, 200, 400, and 800 mM). 

### 3.10. Statistical Analyses

All the experiments were carried out in triplicate to obtain mean values and standard deviations (SD), and the data are presented as means ± SD. The differences in the physicochemical properties, functional attributes, and chemical composition of each IDF sample were computed using SPSS Statistics 26.0 (Chicago, IL, USA), applying Duncan’s multiple range test (*p* < 0.05). Ultimately, the data are visually presented through Origin 2018 (Northampton, MA, USA).

## 4. Conclusions

*Rosa roxburghii* Tratt pomace offers a versatile raw material for the development of numerous food products. This study suggests that the combination of ultrasound and cellulase treatment is an effective modification method in enhancing the quality and functionality of IDF from RRTP. This modification treatment can increase the SDF content and enhance the physicochemical and functional properties of IDF. The modified IDF has the potential to be developed as a functional food ingredient, exhibiting improved water-holding capacity, oil-binding capacity, swelling capacity, nitrite ion adsorption capacity, cholesterol adsorption capacity, and bile salt adsorption capacity. Owing to its enhanced health benefits and functional properties, further research and exploration of the potential applications of modified IDF in the food and pharmaceutical industries are justified.

## Figures and Tables

**Table 1:** The chemical composition of RIDF, U-RIDF, C-RIDF, and UC-RIDF *.

	RIDF	U−RIDF	C−RIDF	UC−RIDF
Fat (%)	1.62 ± 0.03 ^a^	1.56 ± 0.03 ^a^	1.26 ± 0.10 ^b^	1.08 ± 0.05 ^c^
Protein (%)	2.45 ± 0.11 ^a^	2.31 ± 0.05 ^a^	2.40 ± 0.05 ^a^	1.74 ± 0.21 ^b^
Ash (%)	3.84 ± 0.05 ^b^	4.13 ± 0.05 ^a^	3.88 ± 0.07 ^b^	3.76 ± 0.02 ^b^
SDF (%)	3.92 ± 0.01 ^d^	7.86 ± 0.02 ^c^	8.23 ± 0.01 ^b^	9.15 ± 0.02 ^a^
IDF (%)	87.47 ± 0.02 ^a^	81.61 ± 0.01 ^c^	82.58 ± 0.02 ^b^	81.34 ± 0.17 ^b^
TDF (%)	91.39 ± 0.04 ^a^	89.47 ± 0.02 ^d^	90.82 ± 0.03 ^b^	90.49 ± 0.16 ^c^

* Values are expressed as the mean ± SDs (*n* = 3). Different lowercase letters indicate remarkable differences between groups at *p* < 0.05. RIDF: *Rosa roxburghii* Tratt pomace insoluble dietary fiber; U−RIDF: RIDF modified by ultrasound; C−RIDF: RIDF modified by cellulase; UC−RIDF: RIDF modified by ultrasound-assisted cellulase; SDF: soluble dietary fiber; IDF: insoluble dietary fiber; TDF: total dietary fiber.

**Table 2:** Water holding capacity (WHC), oil holding capacity (OHC), and swelling capacity (SC) of RIDF, U−RIDF, C−RIDF, and UC−RIDF *.

	WHC (g/g)	OHC (g/g)	SC (mL/g)
RIDF	10.21 ± 0.19 ^d^	5.96 ± 0.03 ^c^	5.42 ± 0.05 ^c^
U−RIDF	10.67 ± 0.28 ^c^	10.11 ± 0.09 ^b^	6.93 ± 0.07 ^c^
C−RIDF	12.1 ± 0.1 ^a^	11.11 ± 0.07 ^a^	7.93 ± 0.08 ^b^
UC−RIDF	11.44 ± 0.13 ^b^	11.01 ± 0.09 ^a^	10.37 ± 0.11 ^a^

* Values are expressed as the mean ± SDs (*n* = 3). Different lowercase letters indicate remarkable differences between groups at *p* < 0.05. RIDF: *Rosa roxburghii* Tratt pomace insoluble dietary fiber; U−RIDF: RIDF modified by ultrasound; C−RIDF: RIDF modified by cellulase; UC−RIDF: RIDF modified by ultrasound-assisted cellulase.

**Table 3:** TPC and antioxidant activities of IDF, U−RIDF, C−RIDF, and UC−RIDF *.

	FPC (mg GAE/100 g)	BPC (mg GAE/100 g)	TPC (mg GAE/100 g)	DPPH (μmol TE/g)	ABTS (μmol TE/g)
RIDF	180.19 ± 3.46 ^a^	344.18 ± 4.00 ^a^	524.37 ± 2.00 ^a^	41.01 ± 0.32 ^a^	33.42 ± 0.99 ^a^
U−RIDF	98.19 ± 2.00 ^b^	323.48 ± 3.05 ^b^	421.00 ± 3.05 ^b^	33.82 ± 0.37 ^b^	27.12 ± 0.30 ^b^
C−RIDF	86.18 ± 2.00 ^c^	310.79 ± 3.05 ^c^	396.98 ± 4.16 ^c^	33.72 ± 0.98 ^b^	24.25 ± 0.54 ^c^
UC−RIDF	71.45 ± 1.15 ^d^	279.88 ± 2.00 ^d^	351.33 ± 1.15 ^d^	28.68 ± 0.19 ^c^	20.43 ± 0.94 ^d^

* Values are expressed as means ± SDs (*n* = 3). Different lowercase letters indicate remarkable differences between groups at *p* < 0.05. RIDF: *Rosa roxburghii* Tratt pomace insoluble dietary fiber; U-RIDF: RIDF modified by ultrasound; C−RIDF: RIDF modified by cellulase; UC−RIDF: RIDF modified by ultrasound−assisted cellulase.

## Data Availability

Data are contained in the article.

## Abbreviations

RRTP: *Rosa roxburghii* Tratt pomace; RIDF: *Rosa roxburghii* Tratt pomace insoluble dietary fiber; U–RIDF: RIDF modified by ultrasound; C–RIDF: RIDF modified by cellulase; UC–RIDF: RIDF modified by ultrasound-assisted cellulase; DF: dietary fiber; IDF: insoluble dietary fiber; SDF: soluble dietary fiber; WHC: water–holding capacity; OHC: oil-binding capacity; SC: swelling capacity; NIAC: nitrite ion adsorption capacity; CAC: cholesterol adsorption capacity; BSAC: bile salt adsorption capacity; TPC: total phenolic content; BPC: bound phenolic content; FPC: free phenolic content.
